# Nueve años de tendencia en la resistencia a ciprofloxacina por *Escherichia coli*: estudio transversal en un hospital de Colombia

**DOI:** 10.1590/0102-311XES031723

**Published:** 2024-08-26

**Authors:** Emy Shilena Torres Villalobos, José Alberto Mendivil De la Ossa, Yolima Pertuz Meza, Andres Camilo Rojas Gulloso

**Affiliations:** 1 Universidad Cooperativa de Colombia, Santa Marta, Colombia.

**Keywords:** Escherichia coli, Ciprofloxacina, Farmacorresistencia Bacteriana, Vigilancia Epidemiológica, Estudios Transversales, Escherichia coli, Ciprofloxacin, Bacterial Drug Resistence, Epidemiologic Surveillance, Cross-sectional Studies, Escherichia coli, Ciprofloxacina, Farmacorresistência Bacteriana, Vigilância Epidemiológica, Estudos Transversais

## Abstract

La ciprofloxacina es un antibiótico de importancia crítica para la salud humana. El aumento de la resistencia de *Escherichia coli* a ciprofloxacina es un problema de salud pública global por su importancia en el tratamiento de infecciones urinarias complicadas y otras infecciones graves; sin embargo, su prescripción es alta en el caribe colombiano. El objetivo fue determinar la tendencia de resistencia de *E. coli* a ciprofloxacina en un hospital colombiano de alta complejidad. A partir de reportes de antibiogramas, los aislados fueron categorizados según los criterios del Instituto de Normas Clínicas y de Laboratorio de los Estados Unidos para cada año estudiado; se calcularon proporciones y se exploraron diferencias en la sensibilidad con pruebas χ^2^. Se utilizó la prueba de Cochran-Armitage para evaluar la tendencia de la resistencia. Valores de p ≤ 0,05 se consideraron significativos. Se analizaron 6.848 aislados, encontrándose una resistencia de 49,31%. Según el origen, la resistencia más alta fue en muestras comunitarias (51,96% - IC95%: 50,51; 53,41), y por tipo de muestra, en piel y tejidos (61,76% - IC95%: 56,96; 66,35) y orina (48,97% - IC95%: 47,71; 50,23). Se halló una tendencia al aumento en la resistencia por año (p < 0,0001), en muestras comunitarias (p = 0,0002) y en orina (p < 0,0001). La resistencia a ciprofloxacina es alta y tiende al aumento en comunidad y en orina, superando el límite establecido para su uso a nivel ambulatorio, lo que es preocupante por la alta prescripción de fluoroquinolonas en la localidad.

## Abstract (en)

Ciprofloxacin is a critically important antibiotic for human health. The increase of *Escherichia coli* resistance to ciprofloxacin is a global public health problem due to its importance in the treatment of complicated urinary tract infections and other serious infections; however, its prescription is high in the Colombian Caribbean. The objective was to determine the resistance trend of *E. coli* to ciprofloxacin in a Colombian hospital of high complexity. From antibiogram reports, isolates were categorized according to Clinical and Laboratory Standards Institute criteria for each year studied; proportions were calculated and differences in sensitivity were explored using the χ^2^ test. The Cochran-Armitage test was used to evaluate the resistance trend. Significance was considered when p-value ≤ 0.05. In total, 6,848 isolates were analyzed, and 49.31% resistance was found. According to origin, the highest resistance was in community samples (51.96% - 95%CI: 50.51; 53.41), and by type of sample, in skin and tissues (61.76% - 95%CI: 56.96; 66.35) and urine (48.97% - 95%CI: 47.71; 50.23). Increasing trends were observed for resistance per year (p < 0.0001), community samples (p = 0.0002) and urine (p < 0.0001). Resistance to ciprofloxacin is high and tends to increase in the community and in urine, exceeding the limit established for its use at the ambulatory level, which is of concern due to the high prescription of fluoroquinolones in the locality.

## Resumo

A ciprofloxacina é um antibiótico extremamente importante para a saúde humana. O aumento da resistência da *Escherichia coli* à ciprofloxacina é um problema de saúde pública global devido à sua importância no tratamento de infecções complicadas do trato urinário e outras infecções graves; no entanto, sua prescrição é alta no caribe colombiano. O objetivo foi determinar a tendência de resistência da *E. coli* à ciprofloxacina em um hospital colombiano de alta complexidade. A partir de relatórios de antibiogramas, os isolados foram categorizados de acordo com os critérios do Instituto de Padrões Clínicos e Laboratoriais dos Estados Unidos para cada ano estudado; as proporções foram calculadas e as diferenças de sensibilidade foram exploradas com os testes χ^2^. O teste de Cochran-Armitage foi usado para avaliar a tendência de resistência. Os valores de p ≤ 0,05 foram considerados significativos. Um total de 6.848 isolados foi testado e foi encontrada uma taxa de resistência de 49,31%. Por origem, a resistência foi mais alta em amostras comunitárias (51,96% - IC95%: 50,51; 53,41) e, por tipo de amostra, em pele e tecidos (61,76% - IC95%: 56,96; 66,35) e urina (48,97% - IC95%: 47,71; 50,23). Foi encontrada uma tendência de aumento na resistência por ano (p < 0,0001), em amostras da comunidade (p = 0,0002) e na urina (p < 0,0001). A resistência à ciprofloxacina é alta e tende a aumentar na comunidade e na urina, excedendo o limite estabelecido para uso ambulatorial, o que é preocupante, dada a alta prescrição de fluoroquinolonas na localidade.

## Introducción

La Organización Mundial de la Salud (OMS) incluyó a las quinolonas en el grupo de las cinco categorías de antimicrobianos críticos y prioritarios para la medicina humana, porque constituyen los pocos, o quizás el único tratamiento disponible, en infecciones complicadas en pacientes con morbilidades graves; además, tienen una alta frecuencia de uso clínico y esto favorece la selección de bacterias resistentes a partir de fuentes no humanas ^1^. La ciprofloxacina es una fluoroquinolona semisintética de amplio espectro, recomendada desde 1991 en las guías clínicas de enfermedades infecciosas de los Estados Unidos y Europa para el tratamiento de las infecciones del tracto urinario no complicadas ^2^; debido a su costo relativamente bajo y a sus especiales características farmacológicas: facilidad de administración oral o intravenosa, capacidad para alcanzar concentraciones terapéuticas en la mayoría de los tejidos y baja toxicidad ^3^. En 1995 representó el 11% de la prescripción médica mundial y en 2001 llegó a ser el antimicrobiano más vendido para el tratamiento de las infecciones del tracto urinario, principalmente en países donde la resistencia de trimetoprim-sulfametozaxol se mantenía entre el 10% y 20% ^4^. Su uso indiscriminado condicionó la resistencia, por lo que en 2010 asociaciones internacionales recomendaron evitarla como tratamiento de primera línea para manejar infecciones del tracto urinario no complicadas, y reservarla solo para infecciones graves. Además, su uso a nivel ambulatorio es aprobado solo cuando la resistencia local es inferior al 10% ^5^. El problema de la resistencia a la ciprofloxacina está dado también por múltiples factores diferentes al uso clínico, como la utilización en medicina veterinaria y en la industria pecuaria, como antibiótico y promotor de crecimiento ^6^. Sin embargo, la automedicación, la venta libre y el uso indiscriminado en tratamientos empíricos por el personal sanitario contribuyen mayoritariamente a la resistencia ^7^.

*Escherichia coli* fue incluida por la OMS en 2014 en la lista de los microorganismos prioritarios para la vigilancia de resistencia antimicrobiana, especialmente a fluoroquinolonas y cefalosporinas de tercera generación. Aunque forma parte de la flora intestinal de humanos y animales, no solo es la causa más frecuente de infecciones del tracto urinario en ambientes comunitarios e intrahospitalarios, sino de bacteriemias en cualquier edad; además, está asociada a infecciones abdominales, de piel y tejidos blandos, y meningitis, esta última en neonatos. También es el principal agente etiológico en infecciones transmitidas por alimentos ^8^.

La resistencia de *E. coli* a ciprofloxacina en infecciones del tracto urinario ha incrementado sustancialmente a nivel global en las últimas tres décadas; en 1990 la resistencia era < 1%; pero aumentó de 1,2% a 2,5% entre 1998 y 2001, y para 2009 era cercana al 20% ^9^. Como ejemplo, en Suiza, la resistencia creció desde 1,8% a 15,9% en 10 años ^10^. De 2010 en adelante, la resistencia en algunas regiones ha alcanzado más de 50%. Particularmente, en la región de Asia y el Pacífico, la resistencia aumentó en 24,8 puntos, entre 2010 y 2013, alcanzando 68,7% al final de este período ^11^; en India el aumento fue de 26,5 puntos entre 2011 y 2017 ^12^.

Estudios de otras muestras clínicas, como hemáticas, intraabdominales y respiratorias; muestran también incremento de la resistencia en el tiempo, lo que constituye un motivo de preocupación por las limitaciones terapéuticas actuales. En Estados Unidos, la resistencia de *E. coli* a ciprofloxacina en hemocultivos entre 1998 y 2007 pasó de 0% a 12% ^13^; en Grecia, la resistencia de hemocultivos en salas de hospitalización aumentó en 6,6 puntos, con 25,6% en 2010 y 32,2% en 2017 ^14^. En la India, la resistencia en infecciones intraabdominales y en infecciones respiratorias en cepas negativas para betalactamasas de espectro extendido (ESBL) en 2014 fue de 58% ^15^.

El informe del Sistema Mundial de Vigilancia de la Resistencia y el Uso de Antimicrobianos (*Global Antimicrobial Resistance and Use Surveillance Systems* - GLASS) en 2021 mostró que la resistencia a ciprofloxacina en orina y en sangre fue dinámica entre los países participantes. En la orina, la resistencia varió desde 8,4% en Finlandia a 77,8% en Egipto; sin embargo, el 50% de los países registraron resistencias entre 25,6% y 57,5%; en muestras de sangre también se encontraron grandes diferencias, desde 11,3% en Noruega hasta 81,6% en Pakistán, con el 50% de los datos en un rango de resistencia de 24,9% y 48,7% ^16^ ^,^ ^17^.

La resistencia a este antimicrobiano también varía entre hospitales de un mismo país. En centros clínicos de Grecia para el 2012 oscilaba entre 5,6% y 49,5% ^18^. En África, también, la resistencia de E. coli uropatógena comunitaria en los tres centros de salud más grandes de Sudáfrica fue de 18,5% ^19^, pero en Ghana, de 62,3% ^20^. En Latinoamérica se halló que, en Brasil, el 18,8% de los aislados de *E. coli* en pacientes ambulatorios de un hospital de tercer nivel en Brasilia fueron resistentes ^21^, mientras que en Nicaragua se reportó un 31,8%, y en regiones de escasos recursos en Bolivia la resistencia en comunidad alcanzó 44% ^22^.

En Colombia el panorama es similar; varios estudios han reportaron incremento de la resistencia a ciprofloxacina, pero también su disminución asociada a la implementación de programas de control de infecciones y de optimización del uso de antibióticos (PROA). Entre 2001 y 2003, la resistencia de *E. coli* a ciprofloxacina en 14 unidades de cuidados intensivos (UCI) en Bogotá fue superior al 20% ^23^. En 2006, la resistencia en UCI aumentó al 25%; sin embargo, en las salas de hospitalización se observó una reducción significativa de la resistencia (31% al 25%) ^24^. De 2007 a 2009, un estudio con 79 hospitales reportó una disminución considerable de la resistencia, del 27% al 21% en hospitalización, y del 28,2% al 25,7% en UCI ^25^. En otra área de Colombia se realizó una investigación entre 2007 y 2012, en 23 hospitales, y aunque se reportó una resistencia general (hospitalización y UCI) del 39,4% en el primer año, al final del período hubo una reducción relevante de la resistencia (31,9%) ^26^. Un estudio reciente entre 2018 y 2021 reportó que, en comparación con otros antimicrobianos tamizados, la resistencia de *E. coli* a ciprofloxacina fue la más alta, con una media del 36,5% (40%-38%) en salas de hospitalización y del 37% (34%-39%) en UCI ^27^. Existen reportes en donde la resistencia ha alcanzado el 84%, como se encontró en un estudio realizado en 2016, en infecciones abdominales de hospitales de cuatro ciudades colombianas ^28^. Lo anterior muestra el incremento gradual de la resistencia y lo dinámico que es este fenómeno; a pesar de los éxitos temporales en la disminución de la propagación de cepas resistentes.

La prescripción de fluoroquinolonas en Colombia ha sido bastante alta; un estudio en 2020, acerca de los patrones de prescripción de antibióticos determinó que las fluoroquinolonas son la tercera categoría de antimicrobianos más prescritos (10%), después de penicilinas (38,6%) y cefalosporinas (30,2%). En la región Caribe, zona donde se encuentra localizada Santa Marta, objeto de este estudio, la prescripción de fluoroquinolonas, macrólidos y aminoglucósidos fue más común que en las otras regiones geográficas ^29^; particularmente esta ciudad ocupa el sexto lugar en la prescripción de fluoroquinolonas ^30^.

El presente estudio se propuso determinar la tendencia de la resistencia de *E. coli* a ciprofloxacina, por origen y tipo de muestra, en una institución de salud de alta complejidad en Santa Marta en un período de 9 años.

## Materiales y métodos

Se ejecutó un estudio descriptivo, retrospectivo y transversal de la tendencia en la resistencia de *E. coli* a ciprofloxacina entre enero de 2013 y diciembre de 2021, basado en reportes de pruebas de identificación y de susceptibilidad en una institución sanitaria de alta complejidad en Santa Marta. Los registros fueron extraídos del aplicativo Whonet, versión 5.6 (https://whonet.org/software.html), y los reportes anuales fueron exportados y transformados a libros de Microsoft Excel, versión 19 (https://products.office.com/), software en el que se depuraron de las variables que no revestían interés.

La procedencia de las muestras fue recategorizada en: “comunidad”, “hospitalización” y “cuidados intensivos”. Las primeras incluían todas las muestras de urgencias, consulta externa y pacientes particulares; las segundas, todos los servicios de hospitalización y quirófanos, y las últimas incluían las unidades de cuidados intensivos de adultos, pediátricos y neonatos. Las muestras se recategorizaron, en “respiratorios”, “líquidos”, “sangre”, “orina”, “heces”, y “piel y tejidos”, según su tipo. Se utilizaron los criterios del Instituto de Normas Clínicas y de Laboratorio de los Estados Unidos (*Clinical and Laboratory Standards Institute* - CLSI) para ciprofloxacina avalados antes de 2018 y desde 2019 para clasificar la susceptibilidad en “sensible”, “intermedio” y “resistente”.

Todos los análisis fueron hechos con R, versión 4.2.2 (http://www.r-project.org), y RStudio, versión 2022.12.0+353 (https://posit.co/download/rstudio-desktop/), con múltiples paquetes. Se calcularon las proporciones para variables categóricas con sus intervalos de 95% de confianza (IC95%) en el análisis descriptivo y se obtuvieron diagramas de líneas para evaluar las tendencias. La independencia entre las variables estudiadas y la susceptibilidad de la bacteria fue evaluada con pruebas χ^2^ de Mantel-Hanzel. Además, se evaluó la tendencia en las proporciones de resistencia en los años estudiados a través de una prueba de Cochran-Armitage, para lo que la susceptibilidad fue considerada como “sensible” y “resistente”, dejando en esta última categoría a los aislados que clasificaran en los criterios de “sensibilidad intermedia” y “resistente” en su respectivo año. Se consideró significancia a los valores p ≤ 0,05.

### Aspectos éticos

Este estudio se clasificó como una investigación sin riesgo basado en la *Resolución nº 8.430* de 1993 del Ministerio de Salud de Colombia, no hubo intervenciones, los datos fueron retrospectivos y los pacientes anonimizados. Las convenciones y leyes nacionales e internacionales de la ética de la investigación en humanos fueron respetadas. El estudio fue aprobado por el comité de investigación de la Universidad Cooperativa de Colombia (código INV2625), y la dirección científica de la Clínica La Milagrosa de Santa Marta.

## Resultados

### Muestra y características

Se analizaron 6.848 aislados de *E. coli* en un período de 9 años entre 2013 y 2021, con una media de 760,8 observaciones anuales. Los años con menor y mayor cantidad de muestras fueron 2018 (342) y 2015 (983), respectivamente. Las muestras más comunes fueron las de orina con 87,9% (6.024) y las de piel y tejidos, 5,9% (408), seguidas de las de sangre, 3,9% (268). Según el ambiente de origen la mayoría fueron comunitarias 66,6% (4.561), seguidas por las de cuidados intensivos con 22,7% (1.555). La prevalencia de resistencia a ciprofloxacina fue del 49,3 % (3.337). La Tabla 1 presenta la descripción de la muestra según estas características.

**Tabla 1:** Descripción de las muestras por año, tipo de muestra y servicio de origen.

	n	%	IC95%
Año			
2013	639	9,3	8,6; 10,0
2014	882	12,8	12,1; 13,7
2015	983	14,4	13,5; 15,2
2016	954	13,9	13,1; 14,7
2017	963	14,0	13,2; 14,9
2018	342	4,9	4,5; 5,5
2019	803	11,7	10,9; 12,5
2020	619	9,0	8,3; 9,7
2021	663	9,6	9,0; 10,4
Tipo de muestra			
Heces	72	1,0	0,8; 1,3
Líquidos	37	0,5	0,3; 0,7
Orina	6.024	87,9	87,1; 88,7
Piel y tejidos	408	5,9	5,4; 6,5
Respiratorios	39	0,5	0,4; 0,7
Sangre	268	3,9	3,4; 4,4
Servicio de origen			
Comunidad	4.561	66,6	65,4; 67,7
Cuidados intensivos	1.555	22,7	21,7; 23,7
Hospitalarios	732	10,6	9,9; 11,4

IC95%: intervalo de 95% de confianza.

En la distribución de los tres niveles de susceptibilidad por año, 2014 fue el año de más resistencia documentada con 53,1% (469), seguido de 2020 y 2021 con 52,3% (324) y 52,1% (346), respectivamente (Tabla 2). El resultado de la prueba χ^2^ (Tabla 3) indica que existen diferencias significativas entre las proporciones de las categorías de resistencia entre los 9 años (valor p < 0,001), siendo 2018 el año con menor resistencia hallada, con 42,4 % (145); justamente el año con menor número de muestras (Tabla 2).

**Tabla 2:** Distribución de susceptibilidad por tipo de muestra y por origen de la muestra.

	Perfil de susceptibilidad					
	Sensible		Intermedia		Resistente	
	n (%)	IC95%	n (%)	IC95%	n (%)	IC95%
Año						
2013	299 (46,7)	42,9; 50,6	8 (1,2)	0,6; 2,4	33 (51,9)	48,0; 55,8
2014	411 (46,6)	43,3; 49,9	2 (0,2)	0,0; 0,8	469 (53,1)	49,8; 56,4
2015	509 (51,7)	43,3; 49,9	2 (0,2)	0,0; 0,39	474 (48,2)	45,1; 51,3
2016	501 (52,5)	49,3; 55,6	3 (0,3)	0,1; 0,9	450 (47,1)	44,0; 50,3
2017	515 (53,48)	50,3; 56,6	3 (0,3)	0,1; 0,9	445 (46,2)	43,0; 49,3
2018	197 (57,6)	52,3; 62,7	0 (0)	0,0; 1,1	145 (42,4)	37,2; 47,6
2019	343 (42,7)	39,3; 46,1	68 (8,4)	6,7; 10,6	392 (48,8)	45,3; 52,2
2020	228 (36,8)	33,1; 40,7	67 (10,8)	8,6; 13,5	324 (52,3)	48,4; 56,2
2021	256 (38,6)	34,9; 42,3	61 (9,2)	7,2; 11,6	346 (52,1)	48,3; 55,9
Muestra						
Heces	45 (62,50)	50,9; 72,8	0 (0)	0,0; 5,1	27 (37,5)	27,2; 49,1
Líquidos	24 (64,86)	48,7; 78,17	2 (5,4)	1,5; 17,7	11 (29,7)	17,5; 45,8
Orina	2.889 (47,96)	46,7; 49,2	185 (3,1)	2,6; 3,5	2.950 (48,9)	47,7; 50,2
Piel y tejidos	151 (37,0)	32,5; 41,8	5 (1,2)	0,52; 2,8	252 (61,8)	56,9; 66,4
Respiratorios	19 (48,72)	33,9; 63,8	4 (10,2)	4,1; 23,6	16 (41,0)	27,1; 56,6
Sangre	131 (48,88)	42,9; 54,8	16 (5,9)	3,7; 9,5	121 (45,2)	39,3; 51,1
Servicio de origen						
Comunidad	2.020 (44,2)	45,8; 45,7	171 (3,7)	3,2; 4,3	2.370 (51,9)	50,5; 53,4
Cuidados intensivos	913 (58,7)	56,2; 61,1	15 (0,96)	0,59; 1,5	627 (40,3)	37,9; 42,7
Hospitalarios	326 (44,5)	40,9; 48,1	26 (3,5)	2,4; 5,1	380 (51,9)	48,2; 55,5

IC95%: intervalo de 95% de confianza.

**Tabla 3:** Resultados de las pruebas de independencia y tendencia con relación a la susceptibilidad.

Diferencia de proporciones *	χ^2^	df	Valor de p
Año *vs*. tipo de muestra	161,22	40	< 0,001**
Servicio de origen *vs*. tipo de muestra	1.130,1	10	< 0,001**
Año *vs*. servicio de origen	790,45	16	< 0,001**
Año *vs*. resistencia	459,01	16	< 0,001**
Servicio de origen *vs*. resistencia	114,9	4	< 0,001**
Tipo de muestra *vs*. resistencia	56,02	10	< 0,001**
Patrón de tendencia en el tiempo ***	Z	dim	Valor de p
Por año	5,49	9	< 0,001 **
Por servicio de origen			
Comunitario	3,67	9	< 0,001 **
Unidad de cuidados intensivos	-1,57	9	0,11
Hospitalización	0,09	9	0,92
Por tipo de muestra			
Heces	0,80	9	0,41
Líquidos	-0,36	9	0,71
Sangre	0,87	9	0,37
Orina	-0,36	9	< 0,001 **
Respiratorias	0,64	9	0,51

df: grados de libertad; dim: años incluidos; Z: estadístico Z.

* Pruebas χ^2^ de Pearson;

*** Prueba de Cochran-Armitage;

** Valores p significativos.

Al analizar la resistencia en función del tipo de muestra, aquellas que fueron obtenidas de secreción de piel y tejidos presentaron mayor prevalencia de resistencia 61,8% (252), seguido de la orina 48,9% (2.950) y sangre 45,2% (121), y en menor medida las de líquidos de cavidades 29,7% (11), como se puede observar en la Tabla 2. Estas diferencias fueron significativas (p < 0,001) (Tabla 3).

**Figura 1:**
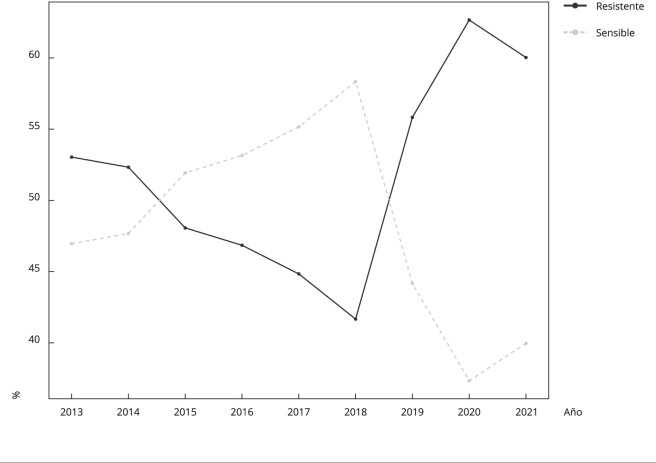
Tendencia de la susceptibilidad por año.

Con relación a lo anterior, la Figura 1, permite ver el comportamiento de la susceptibilidad de *E. coli* a la ciprofloxacina, identificándose a inicios de 2018 un descenso en la cantidad de aislados sensibles con un aumento casi simétrico de los aislados resistentes y con susceptibilidad intermedia, seguido de una meseta en la proporción de resistentes en los dos últimos años que se acompaña de un ligero aumento en los aislados sensibles y con susceptibilidad intermedia.

Por otro lado, en la distribución de la susceptibilidad según el tipo de ambiente o procedencia, los aislados en comunidad y en salas de hospitalización mostraron una proporción similar de resistencia; 52% y 51,9%, respectivamente; mientras que en las unidades de cuidados intensivos la resistencia fue menor (40,3%) (Tabla 2). Adicionalmente, la prueba χ^2^ mostró que existen diferencias significativas en la distribución de estas categorías de resistencia de acuerdo con el tipo de ambiente de donde proceda la muestra (p < 0,001) (Tabla 3).

### Tendencia de la resistencia

El análisis de las tendencias de los grupos de aislados categorizados como sensibles y resistentes mostraron una tendencia significativa p < 0,001 hacia el ascenso entre los períodos observados (Figura 1).

Se aplicó esta prueba a cada categoría de las variables, tipo de muestra y origen de la muestra. Se halló que las únicas muestras que presentaron una tendencia significativa en términos de su resistencia fueron las de orina (valor p < 0,001) en dirección al aumento y las de la comunidad, igualmente en aumento (valor p < 0,001) Tabla 2. En la Tabla 3 se resumen todos los resultados de las pruebas de asociación y tendencia ejecutadas.

## Discusión

Determinamos la prevalencia de *E. coli* resistente a ciprofloxacina en un período de 9 años a partir de 6.848 aislados de comunidad, salas de hospitalización y cuidados intensivos de una institución de salud de referencia en Santa Marta. Nuestros resultados indican que la mayoría de las muestras provienen de fuentes comunitarias (66%), lo cual es compatible con un estudio realizado en Barranquilla, ciudad del Caribe, donde la proporción de aislados en comunidad fue similar (63%); aunque en UCI fue menor (9%), y en hospitalización (28%) ^31^, mayor a lo encontrado aquí (10% y 22,7%, respectivamente).

En cuanto a Colombia, no existen reportes recientes de resistencia a ciprofloxacina en salas de hospitalización y UCI superiores a las encontradas este estudio. En comparación con De la Cadena et al. ^27^, que entre 2018 y 2021 hallaron 36,5% de resistencia en salas de hospitalización y 37% en UCI; encontramos porcentajes superiores, 51,9% y 40,3%, respectivamente. Así también, la resistencia hallada en UCI es ligeramente superior a la informada por el Instituto Nacional de Salud en su Informe de vigilancia de infecciones asociadas a la atención en salud en UCI de adultos (< 40%), en 2018 ^32^.

Al contrastar con otros estudios de años anteriores se observan algunas diferencias en las frecuencias de resistencia según el servicio o ambiente de origen; así, la media de la resistencia en salas de hospitalización y UCI encontrada en este estudio (46,1%) supera el 36,6% encontrado en estos mismos ambientes en 22 hospitales, entre 2007 y 2012 ^26^. De igual manera, la resistencia observada en UCI supera el 30,5% hallado en 2014 para este mismo tipo de servicio en el departamento del Cesar, en la región Caribe ^33^ y el 32,2% reportado en Cali entre 2010 y 2012 ^34^; sin embargo, resulta inferior a lo reportado en UCI de Pereira, región andina, para 2015 (56%) ^35^. En lo referente a muestras comunitarias y de salas de hospitalización, encontramos una resistencia del 51,9% en ambos ambientes, superando el 31,3% de resistencia promedio en muestras comunitarias, y el 49,5% observado en salas de hospitalización en Cali ^34^. Lo anterior da cuenta del carácter heterogéneo de la distribución de la resistencia entre las diferentes regiones de un país y entre servicios hospitalarios y su tendencia creciente a través del tiempo. En Centroamérica, el panorama es más preocupante, se ha reportado hasta 78,3% en UCI en hospitales de México ^36^.

En la distribución de la resistencia por año las cifras ascienden al 50%, particularmente en los dos primeros (51,9% y 53,1%) y los dos últimos años (52,3% y 52,1%); no obstante, cifras superiores se han encontrado, en países árabes, 56,2% ^37^ y Panamá, 77% ^38^. Respecto de la frecuencia por tipo de muestra, nuestros hallazgos son consistentes con lo que plantean la mayoría de las publicaciones en cuanto a que *E. coli* es la bacteria más frecuentemente aislada en orina ^22^; no resultando así con las de piel y tejidos, que fueron las segundas más frecuentemente encontradas, a diferencia de otros estudios donde este lugar lo representan las muestras de sangre ^24^ ^,^ ^26^ ^,^ ^31^. *E. coli* ha sido encontrada como el bacilo gramnegativo multirresistente más frecuente en pie diabético ^39^. En Perú fue el microorganismo más prevalente en infecciones de sitio quirúrgico en Arequipa (40%) ^40^, en otras infecciones ortopédicas nosocomiales se reportó con una frecuencia del 14,3% y con un aumento de la resistencia de 39,5% a 57,6% ^41^. En Colombia, *E. coli* fue la más frecuente en infecciones del pie diabético (22,8%), estando presente en el 41% de infecciones polimicrobianas, con una resistencia a ciprofloxacina del 44,4% ^42^. En nuestro estudio la resistencia es más alta (61,8%), esto puede deberse a que generalmente los aislados de *E. coli* de muestras de piel y tejidos son frecuentemente multirresistentes y se han asociado principalmente a infecciones profundas de heridas contaminadas, siendo también protagonista en las úlceras por presión ^43^. Contrario a lo reportado en este estudio, en Asia Central se halló un aumento en la sensibilidad a ciprofloxacina cercano al 75%, en 2020 ^44^; lo que puede estar relacionado con las regulaciones en el uso de fluoroquinolonas ^45^.

Respecto de la resistencia en orina, existe una amplia documentación; a nivel mundial, la resistencia de *E. coli* uropatógena a ciprofloxacina ha sido mayor en ambientes hospitalarios que en comunidad, reportándose hasta en un 38% ^46^. Específicamente, en Colombia entre 2016 y 2018 se observó una resistencia mayor (49,5%) en un hospital de la región de Caribe ^47^; lo que resulta muy similar a la resistencia encontrada en este estudio (48,9%). En otro hospital de esa misma ciudad la resistencia fue inferior (34,2%); sin embargo, la resistencia a ciprofloxacina en las cepas productoras de betalactamasas de espectro extendido (BLEE) fue del 97,3%; aspecto aún más preocupante por las limitaciones terapéuticas que esto representa ^48^. En países asiáticos como Irán se encontró una resistencia similar (48,4%) ^49^.

De la resistencia de *E. coli* a ciprofloxacina en sangre, estudios en Colombia en 2016 mostraron que 29% de las enterobacterias causantes de bacteriemias son resistentes a quinolonas ^50^. Entre 2018 y 2020 se halló una resistencia de 35,8% en pacientes oncológicos en Bogotá ^51^. En cuanto a Asia se encuentran resistencias superiores, en China se halló 76,8% de aislados resistentes ^52^, lo que resulta muy superior a lo encontrado en este estudio; cabe resaltar que la resistencia encontrada no es despreciable (45,2%), debido a que actualmente no hay reportes de resistencias más altas en Colombia. Se ha documentado que la resistencia de *E. coli* aislada a las fluoroquinolonas tiene un mayor impacto relativo en la mortalidad por bacteriemias, lo que es preocupante debido al aumento en el número de muertes en el mundo por esta causa ^53^.

Hay que precisar que el problema de la resistencia a los antimicrobianos se genera desde diferentes aristas; por tanto, no solo el uso indiscriminado a nivel hospitalario es relevante, sino también la venta libre de fármacos, y la prescripción, incluso por parte de personal de la salud, fenómeno bastante común en países en vía de desarrollo como Colombia, los cuales impactan fuertemente la propagación y crecimiento de la resistencia a nivel comunitario y nosocomial. Esta práctica incorrecta pone de relieve que son un elemento clave adicional que las autoridades en salud pública deberían intervenir específicamente a través de leyes, y que se necesita realizar más estudios destinados a aclarar los posibles impulsores de la dispensación de antibióticos sin receta, por lo que se recomienda un mayor control en la venta y distribución de estos fármacos mediante formulación médica sugerida.

Nuestros hallazgos muestran que la tendencia de la resistencia de *E. coli* a ciprofloxacina es creciente en muestras de orina de pacientes ambulatorios; diferentes estudios dan cuenta de esto, como el realizado en un período de 20 años (2000-2019) que mostró aumento en las tasas de resistencia a lo largo del tiempo ^54^. A esto se suma el problema de la co-resistencia a fluoroquinolonas en aislados BLEE positivos, la cual ha aumentado significativamente a partir del 2000; en Colombia, entre 2005 y 2009 fue de 76,6% ^55^; ya para 2016 el 88,8% de las cepas productoras de BLEE causantes de infecciones de tracto urinario comunitarias en Colombia eran resistentes a ciprofloxacina ^56^.

Santa Marta se identificó como una ciudad con alta prescripción de fluoroquinolonas ^30^, por lo que el aumento de la resistencia en el tiempo en entornos comunitarios podría relacionarse con este factor. Diferentes estudios han demostrado una fuerte correlación entre el consumo de fluoroquinolonas y el aumento de las tasas de resistencia en *E. coli* ^9^ ^,^ ^57^ ^,^ ^58^. Se ha evidenciado el efecto del uso de la ciprofloxacina en la generación de uropatógenos resistentes, Vellinga et al. ^59^ observaron que la resistencia a la ciprofloxacina fue mayor en aislados de pacientes de consultorios donde formulaban 10 prescripciones mensuales (5,5%) *vs*. una mensual 3%. Yang et al. ^58^ asociaron un uso mayor de fluoroquinolonas con un mayor riesgo de adquirir bacterias resistentes, independientemente del historial personal de consumo de antibióticos y de otros factores de riesgo conocidos.

Es de resaltar que las guías para el manejo de infecciones urinarias en Estados Unidos y Europa recomiendan evitar el uso de las fluoroquinolonas, a menos que no existan otras alternativas terapéuticas disponibles. Si bien es cierto, la guía colombiana para la infección de vías urinarias no complicadas, emitida en 2015, no recomendó la ciprofloxacina como tratamiento empírico para las cistitis agudas, ni para las infecciones del tracto urinario que requerían hospitalización; sí la sugirió en las pielonefritis tratadas ambulatoriamente según la sensibilidad del aislado, y en casos sintomáticos de forma excepcional ^60^. Para ese año la resistencia local alcanzaba el 25% ^60^, más del 10% que algunas guías internacionales colocaban como límite para evitar su prescripción, lo que puede asociarse con el aumento de la resistencia en los últimos años.

Recientemente, en 2023, la guía de infecciones urinarias no complicadas no recomendó las fluoroquinolonas como tratamiento empírico, incluso para las infecciones urinarias complicadas, ya que se ha demostrado mayor eficacia clínica y menos efectos adversos con otros antimicrobianos ^61^. La guía está recientemente emitida y nuestros resultados incluyen 9 años atrás, por lo que la tendencia creciente en la resistencia a ciprofloxacina puede estar impulsada por su uso en condiciones clínicas para las que sí estaba aprobada anteriormente, como la pielonefritis complicada y tratamientos de segunda línea para las cistitis, como muestra un estudio en el que el 75% de las prescripciones de fluoroquinolonas se encontraron ajustadas a estas recomendaciones ^30^.

Teniendo en cuenta que en 2021 la OMS incluyó a la ciprofloxacina en el grupo de antibióticos bajo vigilancia dentro de la clasificación AWaRe (acceso, vigilancia y reserva) ^62^, por sus siglas en inglés; sugerimos según los resultados de este estudio que la ciprofloxacina, como otras fluoroquinolonas, no se consideren en el tratamiento de infecciones urinarias, aún sí los aislados muestran susceptibilidad; a menos que no existan otras posibilidades de tratamiento, tal como plantean algunas directrices internacionales.

El problema de la resistencia a los antimicrobianos es complejo; por esto, no solo el uso indiscriminado a nivel hospitalario es relevante sino la venta libre motivada por la autoprescripción; fenómeno común en países en desarrollo como Colombia, donde, pese a existir una normatividad que regula el expendio de medicamentos bajo formula médica, el 80% de las farmacias muestreadas en ciudades como Bogotá, violaban esta reglamentación y el 56% había admitió haber comprado antibióticos sin una prescripción médica, lo cual impacta fuertemente en la propagación y crecimiento de la resistencia a nivel comunitario y nosocomial, debido a la fuerte correlación entre el alto consumo de ciprofloxacina y las tasas de resistencia ^58^. Esta práctica incorrecta destaca la necesidad de implementar una normatividad específica para la dispensación de antibióticos, especialmente los listados por la OMS, con el fin de abordar, de manera estricta la fiscalización de la venta prohibiendo su comercialización en establecimientos farmacéuticos minoristas; y de promover campañas educativas para crear conciencia acerca del impacto de la resistencia antimicrobiana en la salud pública; así como de realizar más estudios para aclarar los impulsores de la dispensación de antibióticos sin receta.

La alta tendencia a la resistencia encontrada en muestras de origen comunitario evidencia un importante hallazgo para Santa Marta porque sugiere una probabilidad de resistencia alta a nivel local, mostrando la necesidad de estudios para determinar su prevalencia, y consecuentemente, emprender una vigilancia integral y rutinaria de la prescripción y consumo de este antimicrobiano desde la óptica de “una salud” que involucre todos los actores que contribuyen al problema con el fin de contener la resistencia y optimizar su uso en todos los ámbitos posibles.

## Limitaciones

El no disponer de datos clínicos de los pacientes es una limitación importante de este estudio, por otro lado, que los resultados de identificación y de sensibilidad como en cualquier caso podrían estar afectados por las prácticas propias del laboratorio para la toma y el procesamiento de muestras, lo cual no es controlado por los investigadores. Adicionalmente, no se pudo establecer una diferencia entre los aislados que provienen de infecciones de aquellos que son producto de una colonización o contaminación, pues, esta diferenciación clínica no se halla en los reportes de identificación y sensibilidad de microorganismos.
